# Association between the cardiometabolic index and osteoporosis: a cross-sectional study of the NHANES

**DOI:** 10.3389/fpubh.2024.1462169

**Published:** 2024-10-03

**Authors:** Deyan Li, Jinli Li, Yijun Li, Wei Dong, Zhuofeng Lin

**Affiliations:** ^1^Department of Orthopedics, Bao’an Clinical Institute of Shantou University Medical College, Shantou, Guangdong, China; ^2^Department of Orthopedics, Shenzhen Bao’an Shiyan People’s Hospital, Shenzhen, Guangdong, China

**Keywords:** cardiometabolic index, osteoporosis, bone density, cross-sectional study, NHANES

## Abstract

**Background:**

The cardiometabolic index (CMI) is a novel metabolic biomarker, but research on its association with osteporosis (OP) is limited. The objective of this research was to clarify the relationship between CMI and OP in the older adult population of the United States.

**Methods:**

This study conducted a cross-sectional analysis using NHANES data 2007–2018 with exclusion of 2011–2012 and 2015–2016 cycles. Logistic regression was used to investigate the relationship between CMI and OP prevalence. Restricted cubic spline curve (RCS) and threshold saturation analyses were performed to explore the nonlinear association between CMI and OP prevalence. Subgroup analyses, sensitivity analyses, and additional analyses were conducted to ensure the robustness and reliability of the findings.

**Results:**

The study included 4,191 participants, revealing that those with OP had significantly lower CMI levels. Logistic regression revealed a strong inverse correlation between Log CMI and OP (OR = 0.72, 95% CI = 0.59–0.88), which persisted after adjusting for covariates. RCS analysis revealed a nonlinear inverse relationship with the critical threshold at CMI = 0.93. Below this threshold, each unit increase in CMI was associated with a 37% reduction in OP prevalence, but changes above this threshold were not significant. Subgroup and sensitivity analyses confirmed the robustness of the findings.

**Conclusion:**

Elevated CMI exhibited a robust inverse correlation with the prevalence of OP in the older adult U.S. population. Maintaining a moderate CMI significantly diminishes the risk of developing OP.

## 1 Introduction

The deterioration of bone microstructure and significant reductions in bone mineral density (BMD) are the hallmarks of osteoporosis (OP), a systemic skeletal disease that leads to a substantially increased risk of fractures and increased bone fragility (1, 2). On a global scale, more than 19.7% of individuals are affected by OP, and the percentages are significantly greater among the older adult population (3). As the global population continues to get older, the occurrence of OP is expected to increase, posing a substantial challenge to public health systems (4). OP substantially elevates the risk of fragility fractures, leading to severe health issues, long-term disability, and a dramatic increase in medical costs and care needs (5, 6). Since OP often progresses without noticeable symptoms in its early stages, it is typically not detected until fractures occur. Hence, early identification and evaluation of risk factors linked to OP are essential for effective clinical interventions and treatment strategies.

Recent studies have established a strong connection between metabolic disorders and OP. Metabolic disorders not only are major risk factors for cardiovascular diseases and diabetes but also negatively impact bone health, contributing to the development and progression of OP (7–9). The cardiometabolic index (CMI) is a newly metabolic indicator that combines the waist-to-height ratio (WHtR) to measure obesity and the levels of triglycerides (TG) and high-density lipoprotein cholesterol (HDL-C) to evaluate lipid health (10). Research indicates that the CMI is a valuable tool for assessing the risk of diabetes-related conditions and is a reliable predictor of diabetes (11). Additionally, CMI has received considerable attention in the evaluation of various disease states (12–14). An observational study revealed a correlation between high CMI and a greater likelihood of experiencing depression (15). This suggests that by effectively controlling dyslipidemia and improving lipid profiles, the risk of developing depression might be reduced. Similarly, another study revealed a direct correlation between CMI and nonalcoholic fatty liver disease (NAFLD) and fibrosis, highlighting the potential of CMI as a reliable indicator of NAFLD and fibrosis (16). Current research has focused on the components of the cardiometabolic syndrome and their impact on bone health, but has often overlooked how composite metrics such as the CMI can provide a more comprehensive risk assessment (17–19). Previous research elucidating an integrated approach to nutritional and physiological assessment has been shown to be effective in predicting health outcomes (20). Based on these findings, the present study aims to fill this research gap by systematically exploring the relationship between CMI as a comprehensive metric and OP prevalence in the older adult population in the U.S. The aim is to provide strong theoretical support and practical guidance for early prevention and intervention strategies for OP.

## 2 Methods

### 2.1 Survey description

The National Health and Nutrition Examination Survey (NHANES) is a study using complex sampling methods representative of the U.S. population to assess nutrition and health. The National Center for Health Statistics (NCHS) Research Ethics Review Board reviews and authorizes research involving human subjects. Participants submitted written informed consent.

### 2.2 Study population

The following were the requirements for this study to be included: (1) were at least 50 years old; (2) had a proximal femur DXA scan and full data available for this scan; and (3) had complete data for waist circumference (WC), body height (BH), TG levels, and HDL-C levels.

### 2.3 Calculation of CMI

Based on anthropometric and laboratory data, the CMI was calculated using a formula that has been established in prior research. The initial calculation of the WHtR involved the use of WC (cm) and BH (cm). The CMI was subsequently calculated by integrating the levels of HDL-C and TG. The formula is as follows:

$$WHtR=\frac{WC\;\left(cm\right)}{BH\;\left(cm\right)}$$

$$CMI=\frac{TG\;\left(mmol/L\right)}{HDL-C\;\left(mmol/L\right)}\times WHtR$$

### 2.4 Definition of osteoporosis

Dual-energy X-ray absorptiometry (DXA) is widely acknowledged as the most reliable method for assessing BMD because of its quickness, ease of use, and low level of radiation exposure. NHANES conducted DXA scans of the proximal femur, which included the entire femur, femoral neck, trochanter, and intertrochanter, at mobile examination centers over the time periods of 2005–2010, 2013–2014, and 2017–2018. The T scores were computed using a comparison group consisting of non-Hispanic white women between the ages of 20 and 29, as recommended by the World Health Organization’s standards (Supplementary Table 1). Individuals whose T scores for total femur BMD, femoral neck BMD, trochanter BMD, or intertrochanter BMD were below −2.5 standard deviations were categorized as having OP (3, 21).

### 2.5 Covariates

This study accounted for multiple covariates, including demographic characteristics, lifestyle factors, health status, and laboratory examination data. The demographic factors included age, sex, race, poverty index ratio (PIR), and education level. Lifestyle variables included smoking status and levels of physical activity. Smoking status was determined by whether an individual had smoked over more than 100 cigarettes in their lifetime. Physical activity was assessed using the Global Physical Activity Questionnaire and quantified in metabolic equivalent (MET) minutes, with levels below less than 600 min per week categorized as inactive. Health status variables included chronic kidney disease, diabetes, and hypertension, as determined by physician diagnosis or self-reports. The laboratory data included the serum calcium, and serum phosphorus concentrations, and levels of the AST, ALT, SCR, and SUA levels.

### 2.6 Statistical analysis

Data from 2007 to 2018 were sourced from the NHANES, excluding the 2011–2012 and 2015–2016 cycles due to missing proximal femur DXA scan data, covering four survey cycles. Participants met the inclusion criteria, and descriptive analyses were performed by OP status. The association between CMI and OP prevalence was analyzed using univariate and multivariate logistic regression models, with CMI dichotomized to strengthen the robustness of the results. RCS and threshold saturation analyses revealed the nonlinear relationship and critical point between CMI and OP. Subgroup analyses considered factors such as age, sex, race, chronic kidney disease, diabetes, and hypertension. Sensitivity analyses confirmed the reliability and stability of the results. Additional studies have explored the correlation between CMI and BMD in various femur locations, supporting the validity of these findings. R software (version 4.2.3) was used for analyses, with a significance level of *p* < 0.05.

## 3 Results

### 3.1 Characteristics of study population

**Figure 1:**
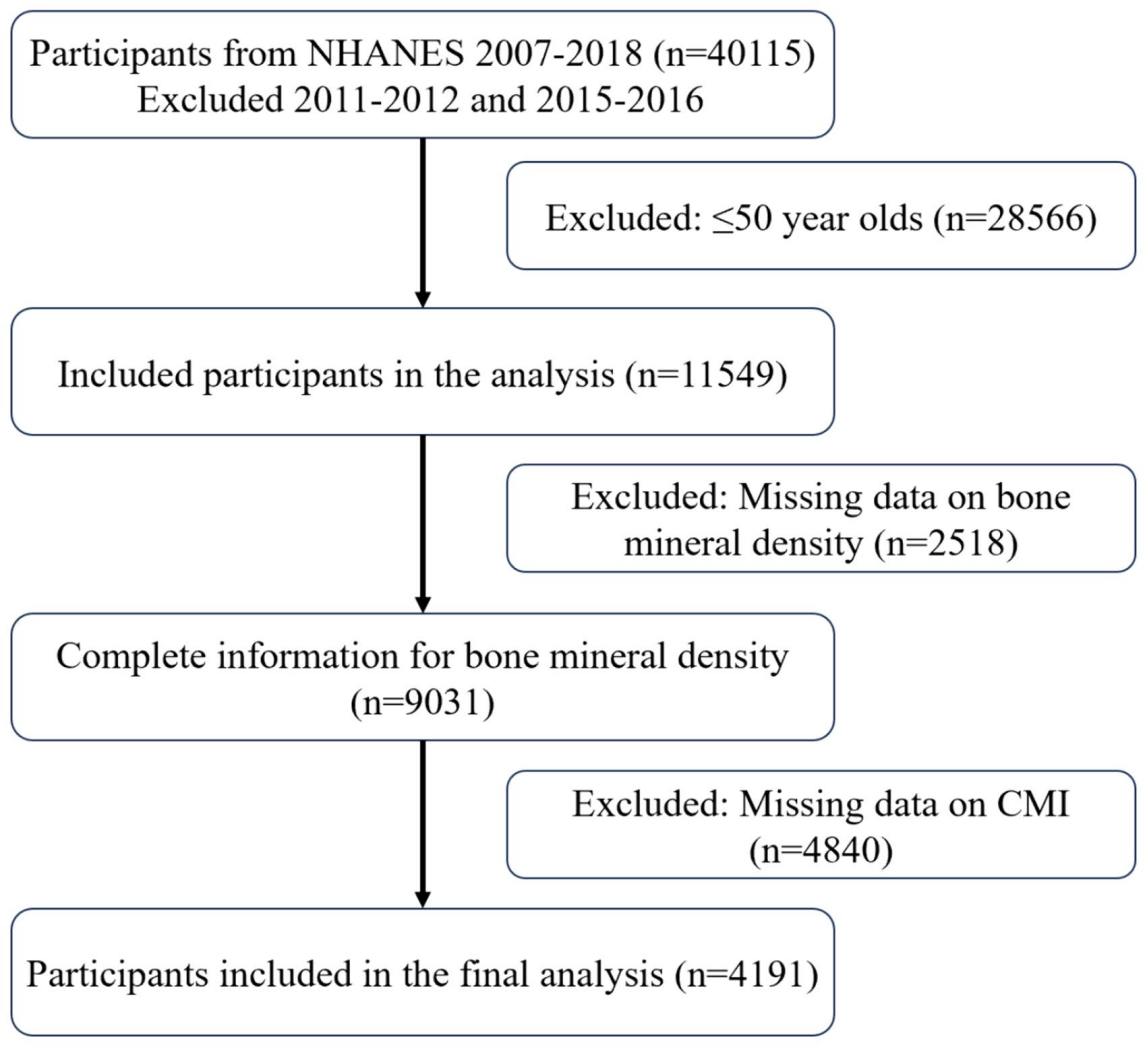
Participants included in the study.

Figure 1 illustrates the selection process, which included 4,191 participants in the cross-sectional analysis. These participants consisted of 3,819 non-OP and 372 OP individuals. The baseline characteristics by OP status are detailed in Table 1. OP patients were typically older adult, had a greater proportion of females, were more likely to be non-Hispanic white, had lower education levels and moderate income levels, and participated in less physical activity. It is important to note that their CMI levels were lower.

**Table 1:** Baseline characteristics of the study population.

Characteristic	Overall	Non-OP	OP	*p* value
*n*	4,191	3,819	372	
Age (%)				
<65	2,311 (55.1)	2,209 (57.8)	102 (27.4)	<0.001
>65	1,880 (44.9)	1,610 (42.2)	270 (72.6)	
Sex (%)				
Female	2,066 (49.3)	1,807 (47.3)	259 (69.6)	<0.001
Male	2,125 (50.7)	2,012 (52.7)	113 (30.4)	
Race (%)				
Mexican American	554 (13.2)	515 (13.5)	39 (10.5)	<0.001
Non-Hispanic Black	806 (19.2)	777 (20.3)	29 (7.8)	
Non-Hispanic White	2,003 (47.8)	1,771 (46.4)	232 (62.4)	
Others	828 (19.8)	756 (19.8)	72 (19.4)	
Education level (%)				
Under high school	1,184 (28.3)	1,055 (27.6)	129 (34.7)	<0.001
High school or equivalent	983 (23.5)	879 (23.0)	104 (28.0)	
Above high school	2,017 (48.1)	1,881 (49.3)	136 (36.6)	
No record	7 (0.2)	4 (0.1)	3 (0.8)	
PIR (%)				
<1	612 (16.3)	542 (15.9)	70 (20.6)	<0.001
1–3	1,666 (44.4)	1,487 (43.6)	179 (52.6)	
>3	1,471 (39.2)	1,380 (40.5)	91 (26.8)	
Activity status (%)				
Active	1,764 (42.1)	1,654 (43.3)	110 (29.6)	<0.001
Inactive	2,427 (57.9)	2,165 (56.7)	262 (70.4)	
Smoke (%)				
No	2,089 (49.8)	1,892 (49.5)	197 (53.0)	0.142
Yes	2,099 (50.1)	1,925 (50.4)	174 (46.8)	
No record	3 (0.1)	2 (0.1)	1 (0.3)	
Hypertension (%)				
No	1,914 (45.7)	1,747 (45.7)	167 (44.9)	0.740
Yes	2,272 (54.2)	2,067 (54.1)	205 (55.1)	
No record	5 (0.1)	5 (0.1)	0 (0.0)	
CKD (%)				
No	3,999 (95.4)	3,662 (95.9)	337 (90.6)	<0.001
Yes	183 (4.4)	149 (3.9)	34 (9.1)	
No record	9 (0.2)	8 (0.2)	1 (0.3)	
Diabetes (%)				
No	3,228 (77.0)	2,928 (76.7)	300 (80.6)	0.157
Yes	807 (19.3)	744 (19.5)	63 (16.9)	
No record	156 (3.7)	147 (3.8)	9 (2.4)	
Total femur BMD [mean (SD)] (gm/cm^2^)	0.92 (0.16)	0.95 (0.15)	0.66 (0.09)	<0.001
Femoral neck BMD [mean (SD)] (gm/cm^2^)	0.76 (0.14)	0.78 (0.13)	0.53 (0.05)	<0.001
Trochanter BMD [mean (SD)] (gm/cm^2^)	0.70 (0.14)	0.72 (0.13)	0.50 (0.08)	<0.001
Intertrochanter BMD [mean (SD)] (gm/cm^2^)	1.10 (0.19)	1.13 (0.17)	0.79 (0.12)	<0.001
Calcium [mean (SD)] (mmol/L)	2.35 (0.09)	2.35 (0.09)	2.34 (0.10)	0.091
Phosphorus [mean (SD)] (mmol/L)	1.17 (0.17)	1.17 (0.17)	1.21 (0.17)	<0.001
ALT [mean (SD)] (IU/L)	23.78 (15.28)	24.09 (15.13)	20.62 (16.44)	<0.001
AST [mean (SD)] (U/L)	25.34 (13.08)	25.40 (13.26)	24.71 (11.06)	0.329
SCR [mean (SD)] (umol/L)	0.95 (0.47)	0.95 (0.47)	0.97 (0.51)	0.283
SUA [mean (SD)] (mg/dL)	5.70 (1.44)	5.73 (1.44)	5.36 (1.46)	<0.001
WC [mean (SD)] (cm)	100.28 (13.79)	101.03 (13.56)	92.50 (13.73)	<0.001
BH [mean (SD)] (cm)	166.16 (10.20)	166.77 (10.09)	159.94 (9.17)	<0.001
TG [mean (SD)] (mg/dL)	1.46 (1.10)	1.47 (1.14)	1.31 (0.66)	0.008
HDL-C [mean (SD)] (mg/dL)	1.43 (0.42)	1.42 (0.42)	1.54 (0.43)	<0.001
CMI [mean (SD)]	0.75 (0.93)	0.77 (0.96)	0.59 (0.50)	0.001

Mean (SD) for continuous variables, % for categorical variables.

ALT, alanine aminotransferase; AST, aspartate aminotransferase; CKD, chronic kidney disease; CMI, cardiometabolic index; PIR, poverty income ratio; SCR, serum creatinine; SUA, serum uric acid; TG, triglyceride; WC, waist circumference.

### 3.2 Association between CMI and proximal femur BMD

To address the left-skewed distribution of CMI, a logarithmic transformation was applied. The results of the logistic regression analysis that examined the relationship between CMI and OP are summarized in Table 2. Model 1 revealed a negative correlation between Log CMI and OP (OR = 0.76, 95% CI = 0.64–0.89). Model 3, which was adjusted for a variety of covariates, demonstrated that a 28% decrease in the prevalence of OP was associated with each unit increase in Log CMI (OR = 0.72, 95% CI = 0.59–0.88). When CMI was dichotomized, a high CMI was significantly negatively associated with OP compared to a low CMI (OR = 0.59, 95% CI = 0.43–0.81). These results indicate a consistent negative association between CMI and OP.

**Table 2:** Relationships between log CMI and OP prevalence.

		Model 1 OR (95%CI) *p* value	Model 2 OR (95%CI) *p* value	Model 3 OR (95%CI) *p* value
OP	log CMI	0.76 (0.64, 0.89) 0.001	0.77 (0.65, 0.92) 0.006	0.72 (0.59, 0.88) 0.002
	Low CMI	[Reference]	[Reference]	[Reference]
	High CMI	0.63 (0.47, 0.85) 0.003	0.65 (0.49, 0.85) 0.002	0.59 (0.43, 0.81) 0.002
	P for trend	0.003	0.002	0.002

CI, Confidence interval; CMI, Cardiometabolic index; OR, Odds ratio; Q, Quartiles.

Model 1, no covariates adjusted; Model 2, adjusted for age, sex, and race; Model 3, Adjusted for age, sex, race, educational level, PIR, calcium, phosphorus, smoking status, hypertension status, CKD status, diabetes status, SCR, BUN, SUA, AST, ALT.

### 3.3 Nonlinear relationship and threshold effect analysis

**Figure 2:**
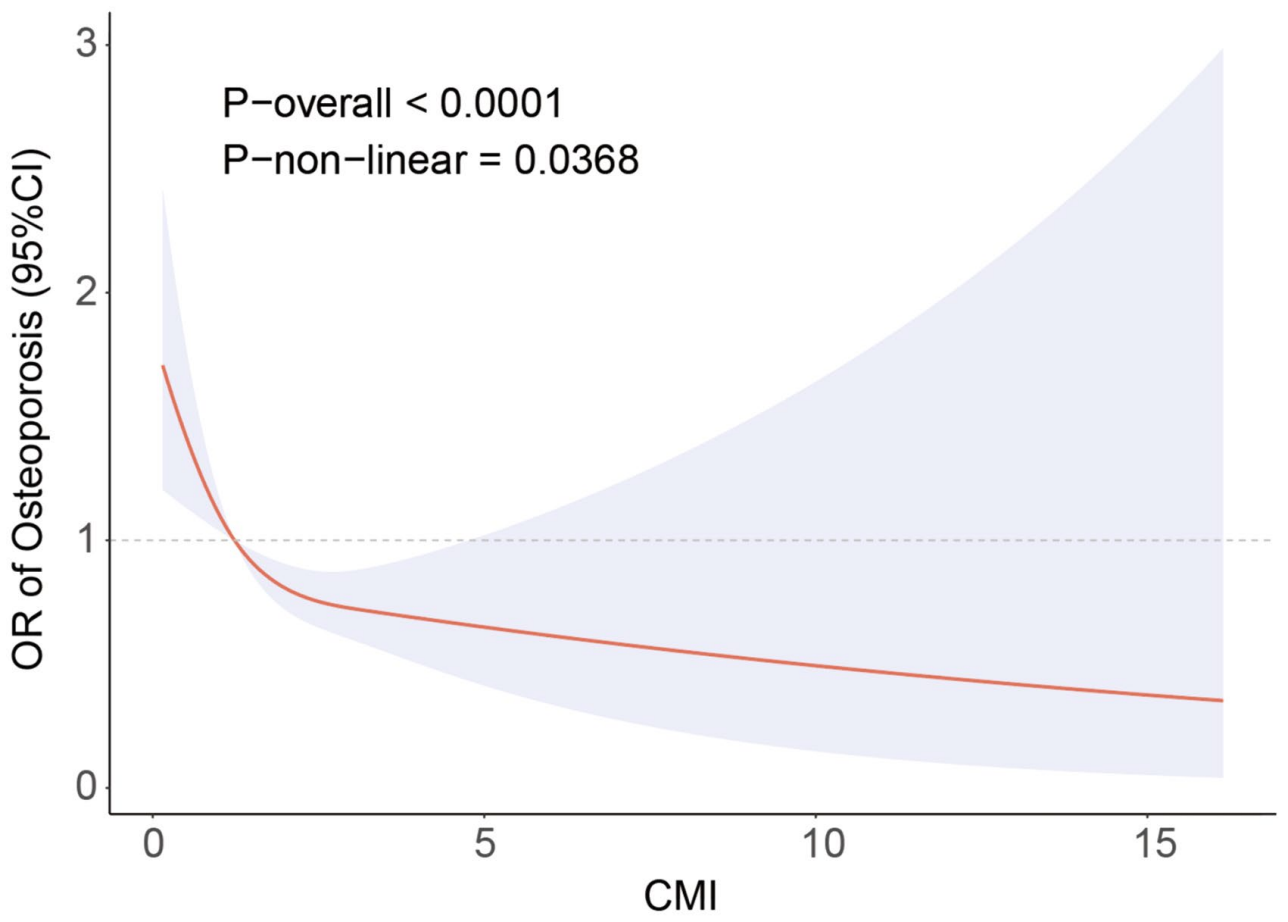
RCS analysis showing the relationship between CMI and OP. Adjusted for age, sex, race, educational level, PIR, calcium, phosphorus, smoking status, hypertension status, CKD status, diabetes status, SCR, BUN, SUA, AST, ALT.

Figure 2 depicts the link between the CMI dosage and the occurrence of OP, as determined by the RCS analysis. The graph shows a nonlinear negative association (P-nonlinear = 0.0368). Threshold effect analysis identified a critical point between CMI and the prevalence of OP at CMI = 0.92. Table 3 shows the relationship between CMI and the prevalence of OP on either side of the critical point. On the left side of the critical point, the prevalence of OP decreases with increasing CMI; on the right side, changes in CMI have no statistically significant impact on the prevalence of OP.

**Table 3:** Analysis of the CMI saturation effect and OP prevalence.

	CMI	OR (95%CI) *p* value
OP	Standard linear model	0.59 (0.44, 0.77) <0.001
	CMI < 0.93	0.35 (0.21, 0.58) <0.001
	CMI > 0.93	0.87 (0.58, 1.14) 0.400
	Log-likelihood ratio test	0.017

CMI, cardiometabolic index; OR, odds ratio; CI, confidence interval.

Adjusted for age, sex, race, educational level, PIR, calcium, phosphorus, smoking status, hypertension status, CKD status, diabetes status, SCR, BUN, SUA, AST, ALT.

### 3.4 Subgroup analysis

**Figure 3:**
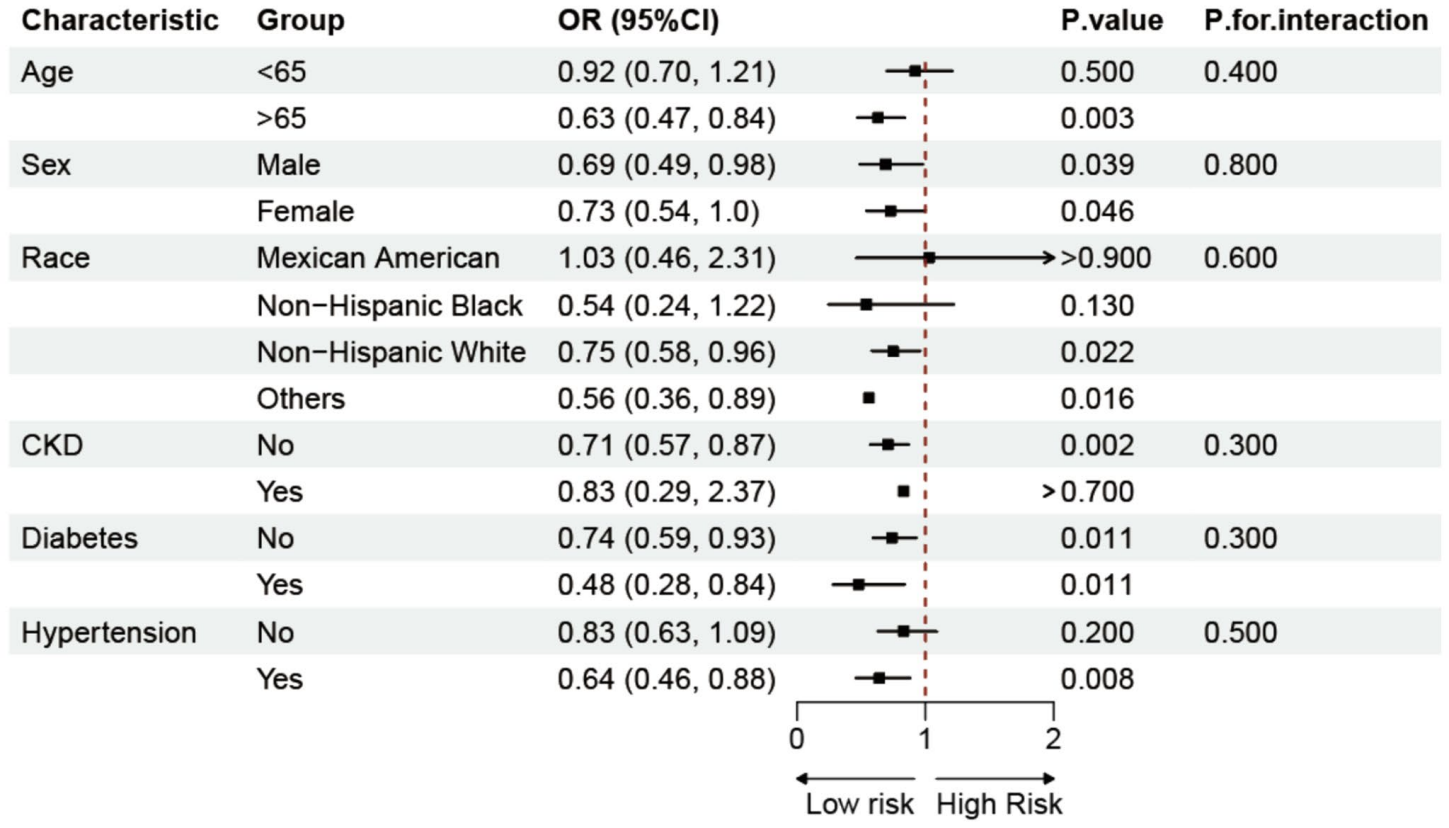
Subgroup analysis of the association between CMI and OP.

To explore the potential influencing factors between CMI and the prevalence of OP, we conducted a subgroup study, combining characteristics such as age, gender, race, chronic kidney disease, diabetes, and hypertension (Figure 3). The results of the analyses showed that the negative association between CMI and OP was more pronounced in people over 65 years of age, whereas the association was not significant in people under 65 years of age. However, the interaction analysis did not find a significant interaction between these factors and the relationship between CMI and OP.

### 3.5 Sensitivity and additional analysis

Sensitivity analyses were implemented to confirm the consistency and robustness of the studies (Supplementary Table 2). After excluding participants with a CMI ± 3 SDs, 4,140 participants remained. After fully adjusting for covariates, the negative association between CMI and the prevalence of OP remained stable. This relationship was also validated when the CMI was dichotomized. Finally, additional analyses were performed. Linear regression analysis demonstrated a consistent positive correlation between CMI and BMD in various regions of the femur, as illustrated in Supplementary Table 3. Supplementary Figures 1–4 demonstrate that the RCS analysis revealed a nonlinear positive relationship between CMI and BMD in several areas of the femur, with the BMD progressively increasing as the CMI increased.

## 4 Discussion

This research analyzed data from 4,191 individuals to explore the relationship between CMI and OP in the older population of the United States. After accounting for several factors, the results confirmed that the inverse association between CMI and OP occurrence persisted. Notably, RCS analysis revealed a nonlinear negative correlation between CMI and OP prevalence, identifying a critical point at CMI = 0.93 through a threshold saturation effect. An increase in CMI within a certain range was linked to a decrease in the occurrence of OP. Subgroup analysis further showed that this negative correlation remained stable across various populations. Finally, sensitivity and supplementary analyses reinforced the robustness and consistency of the findings. These results suggest that within a specific range, CMI may be a protective factor against OP, and further research on CMI could aid in the prevention and delay of OP progression.

In recent years, obesity has grown to be a significant global public health concern. It is associated with higher death rates and an increase in the prevalence of chronic illnesses such diabetes, hypertension, and dyslipidemia (22–24). The connection between obesity and bone metabolism has garnered increasing attention (25). Research has suggested that central obesity, with WC serving as a significant measure, may offer protection against OP. Current research indicates that WC contributes to BMD maintenance as well (18). Another study confirmed that the obesity index (WC, WHtR, BMI) was negatively associated with the risk of OP in older adults (17). People with obesity often have lipid metabolism issues, which are strongly connected to bone health. A study conducted on individuals between the ages of 20 and 59 years revealed a positive correlation between HDL-C levels and BMD in the lumbar spine (26). Subgroup analyses showed that the negative association between CMI and OP was more pronounced in specific populations. This may be due to the fact that older adults are more susceptible to the adverse effects of metabolic abnormalities on bone health, especially when bone loss is more pronounced, potentially making CMI a stronger predictor of OP in these patients (27). However, interaction analyses showed that although CMI was more strongly associated with OP in specific populations, these characteristics did not significantly alter the overall negative correlation between the two, suggesting that CMI, as a composite metabolic index, is generalizable to OP across populations. In summary, CMI as a comprehensive indicator can integrate the roles of multiple metabolic components and simplify the complex process of assessing metabolic syndrome, thus providing a wide range of applications in clinical practice.

Although the precise mechanisms underlying the connection between CMI and OP remain unclear, several hypotheses may explain this link. CMI is a comprehensive indicator to assess obesity and lipid metabolism, which may further respond to OP through adipose tissue and metabolic pathways response pathways. The interplay between obesity and OP is complex and multifaceted, encompassing both protective and risk factors (28, 29). Humans experience the production of cytokines, such as IL-1β, TNF-*α*, and IL-6, from their adipose tissue. These cytokines can activate intracellular signaling pathways, which may result in the loss of bone mass (30). Moreover, disturbances in lipid metabolism play a crucial role in influencing bone health. Obesity-induced dyslipidemia is characterized by elevated levels of lipoproteins rich in TG and reduced levels of HDL-C (24). Oxidized lipids stimulate the transformation of mesenchymal stem cells (MSCs) into adipocytes, but increased levels of cholesterol in the bloodstream suppress the Wnt signaling pathway, which impacts the differentiation of MSCs into osteoblasts (31, 32). Oxidized lipids and cholesterol disrupt the balance between osteoblasts and osteoclasts, affecting bone microenvironment homeostasis and potentially leading to OP (33–35). Moreover, a high TG/HDL-C ratio is a significant marker of insulin resistance and metabolic syndrome and can worsen lipid metabolism disorders, impair osteoblast function, and reduce BMD (36, 37). Our study suggested that CMI is a protective factor against OP, exhibiting a saturation threshold effect. When the CMI falls below the designated threshold, the combined effects of mechanical stress and hormone production have a favorable impact on bones, leading to an increase in BMD. In contrast, the initial positive effects on bone health might be offset by factors like the accumulation of abdominal fat, shifts in body composition, proinflammatory conditions, and insulin resistance once the CMI surpasses this limit. Thus, the relationship between CMI and OP arises from multifactorial and multipathway interactions, necessitating further research to fully understand the biological mechanisms involved in the connection between CMI and bone metabolism.

Our study has several strengths. First, OP is prevalent among older adult individuals, and a national sample of people over 50 years old was selected to address this concern. Second, various covariates were included to control for potential confounding factors. Ultimately, sensitivity and supplemental studies were conducted to verify the strength and reliability of the results. Nevertheless, this research has several limitations. The cross-sectional design restricts the capacity to establish a causal relationship between CMI and OP. In addition, certain variables in this study were collected through self-reported methods, which may have introduced biases such as recall inaccuracies and reporting errors, and these data relied on the memory and honesty of the participants. Despite the sophisticated sampling techniques utilized in this study, the results may not reflect the true situation with complete accuracy. The population analyzed in this study was the older American population, and these results still need to be validated in the future with more extensive surveys of other ethnic populations and age groups. Numerous prospective studies are still needed in the future to further validate the causal association between CMI and OP.

## 5 Conclusion

According to the results of this study, higher CMI is strongly associated with a lower prevalence of OP in the older U.S. population, and maintaining a certain level of CMI may help to reduce the prevalence of OP. This result suggests that accurate management and optimization of CMI may be a key strategy for preventing and reducing the risk of OP.

## Data Availability

The National Health and Nutrition Examination Survey (NHANES) data is a publicly available database for use by personnel worldwide, and all data are available at https://www.cdc.gov/nchs/nhanes/nhanes_products.htm.
