# Supplementary material for: Association between the cardiometabolic index and osteoporosis: a cross-sectional study of the NHANES
Source: Front Public Health. 2024 Oct 3;12:1462169. doi: 10.3389/fpubh.2024.1462169 (PMC11484830; doi:10.3389/fpubh.2024.1462169)
Supplement: Supplementary file 1 [file Table_1.DOCX]

**Supplementary Table 1: Weighted baseline characteristics of included participants**

| Region of interest | Mean (g/cm²) | Standard  deviation(g/cm²) | BMD cutoff values for  Osteoporosis |
| --- | --- | --- | --- |
| Male |  |  |  |
| Total femur | 1.04 | 0.144 | <0.68 |
| Femur neck | 0.93 | 0.137 | <0.59 |
| Trochanter | 0.78 | 0.118 | <0.49 |
| Intertrochanter | 1.21 | 0.172 | <0.78 |
| Female |  |  |  |
| Total femur | 0.94 | 0.122 | <0.64 |
| Femur neck | 0.86 | 0.120 | <0.56 |
| Trochanter | 0.71 | 0.099 | <0.46 |
| Intertrochanter | 1.09 | 0.142 | <0.74 |

WHO diagnostic criteria; Osteoporosis: BMD value < -2.5 SD below mean of young adult reference group.

**Supplementary Table 2 Sensitivity analysis between log CMI and Osteoporosis.（4140）**

|  |  | Model 1  OR (95%CI) P-value | Model 2  OR (95%CI) P-value | Model 3  OR (95%CI) P-value |
| --- | --- | --- | --- | --- |
| Osteoporosis | log CMI | 0.74 (0.62, 0.89) 0.001 | 0.75 (0.62, 0.91) 0.004 | 0.71 (0.58, 0.88) 0.002 |
|  | Low CMI | [Reference] | [Reference] | [Reference] |
|  | High CMI | 0.63 (0.46, 0.84) 0.003 | 0.64 (0.48, 0.84) 0.002 | 0.60 (0.44, 0.83) 0.003 |
|  | P for trend | 0.003 | 0.002 | 0.003 |

CI: Confidence Interval; CMI: Lipid Accumulation Product; OR: Odds Ratio; Q: Quartiles

Model 1: No covariates adjusted

Model 2: Adjusted for age, sex, and race

Model 3: Adjusted for age, sex, race, educational level, PIR, calcium, phosphorus, smoking status, hypertension status, CKD status, diabetes status, SCR, BUN, SUA, AST, ALT.

**Supplementary Table 2 Additional analysis between BRI and BMD.**

|  |  | Model 1  OR (95%CI) P-value | Model 2  OR (95%CI) P-value | Model 3  OR (95%CI) P-value |
| --- | --- | --- | --- | --- |
| Total femur BMD | Log CMI | 0.05 (0.05, 0.06) <0.001 | 0.04 (0.04, 0.05) <0.001 | 0.04 (0.03, 0.04) <0.001 |
|  | Low CMI | [Reference] | [Reference] | [Reference] |
|  | High CMI | 0.07 (0.06, 0.08) <0.001 | 0.06 (0.05, 0.07) <0.001 | 0.04 (0.03, 0.05) <0.001 |
|  | P for trend | <0.001 | <0.001 | <0.001 |
| Femoral neck BMD | Log CMI | 0.03 (0.03, 0.04) <0.001 | 0.03 (0.02, 0.04) <0.001 | 0.02 (0.02, 0.03) <0.001 |
|  | Low CMI | [Reference] | [Reference] | [Reference] |
|  | High CMI | 0.04 (0.03, 0.06) <0.001 | 0.04 (0.03, 0.05) <0.001 | 0.03 (0.02, 0.04) <0.001 |
|  | P for trend | <0.001 | <0.001 | <0.001 |
| Trochanter BMD | Log CMI | 0.04 (0.03, 0.05) <0.001 | 0.03 (0.03, 0.04) <0.001 | 0.03 (0.02, 0.03) <0.001 |
|  | Low CMI | [Reference] | [Reference] | [Reference] |
|  | High CMI | 0.05 (0.04, 0.06) <0.001 | 0.04 (0.03, 0.05) <0.001 | 0.03 (0.02, 0.04) <0.001 |
|  | P for trend | <0.001 | <0.001 | <0.001 |
| Intertrochanter BMD | Log CMI | 0.06 (0.05, 0.07) <0.001 | 0.05 (0.05, 0.06) <0.001 | 0.04 (0.03, 0.05) <0.001 |
|  | Low CMI | [Reference] | [Reference] | [Reference] |
|  | High CMI | 0.08 (0.07, 0.10) <0.001 | 0.07 (0.06, 0.08) <0.001 | 0.05 (0.04, 0.06) <0.001 |
|  | P for trend | <0.001 | <0.001 | <0.001 |

CI: Confidence Interval; CMI: Lipid Accumulation Product; OR: Odds Ratio; Q: Quartiles

Model 1: No covariates adjusted; Model 2: Adjusted for Age, Sex, and Race; Model 3: Adjusted for age, sex, race, educational level, PIR, calcium, phosphorus, smoke, hypertension, CKD, diabetes, SCR, SUA, AST, ALT.


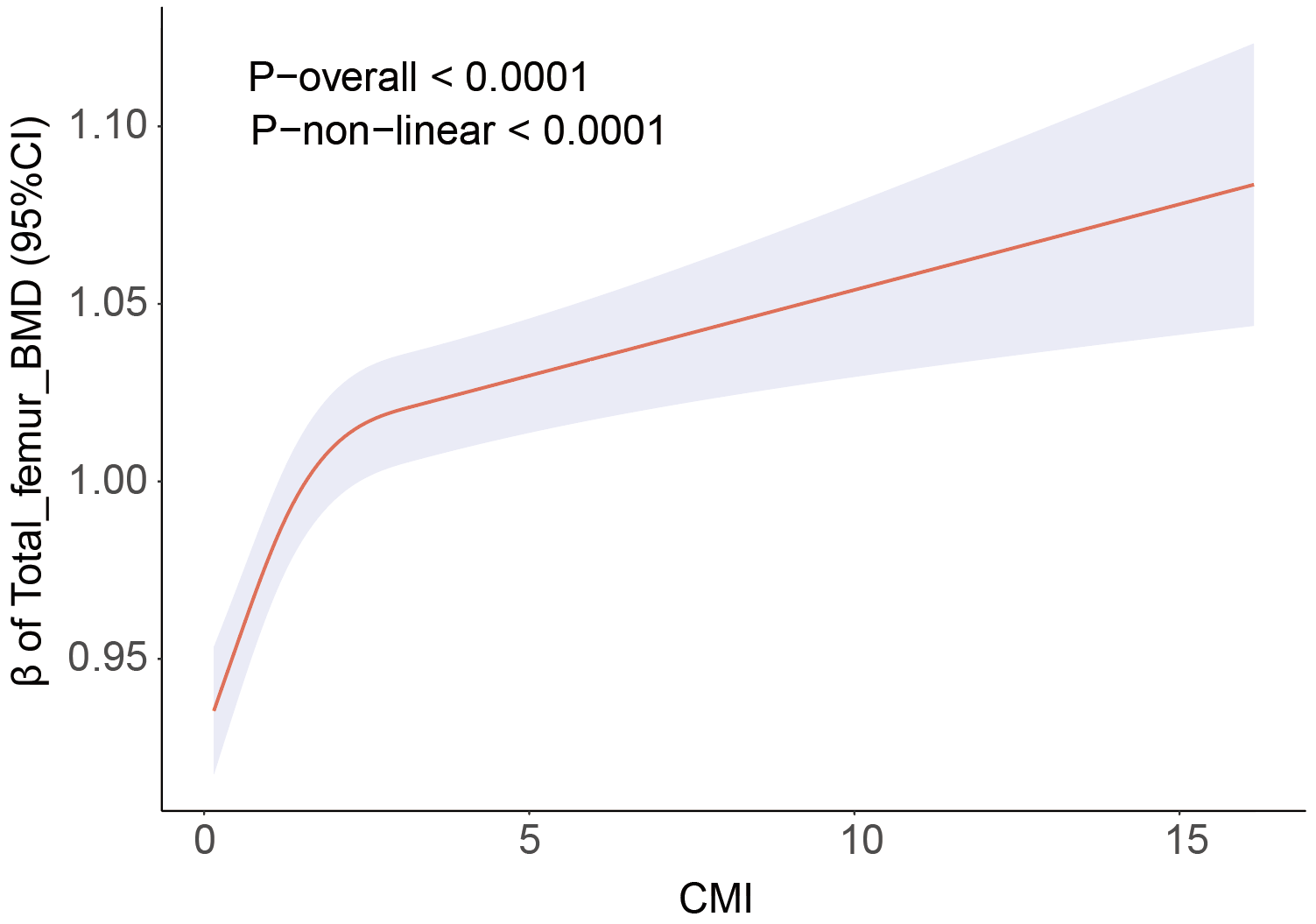


**Supplementary figure 1: RCS analysis fitted the relationship between CMI and Total femur BMD.**

Adjusted for age, sex, race, educational level, PIR, calcium, phosphorus, smoke, hypertension, CKD, diabetes, SCR, SUA, AST, ALT.


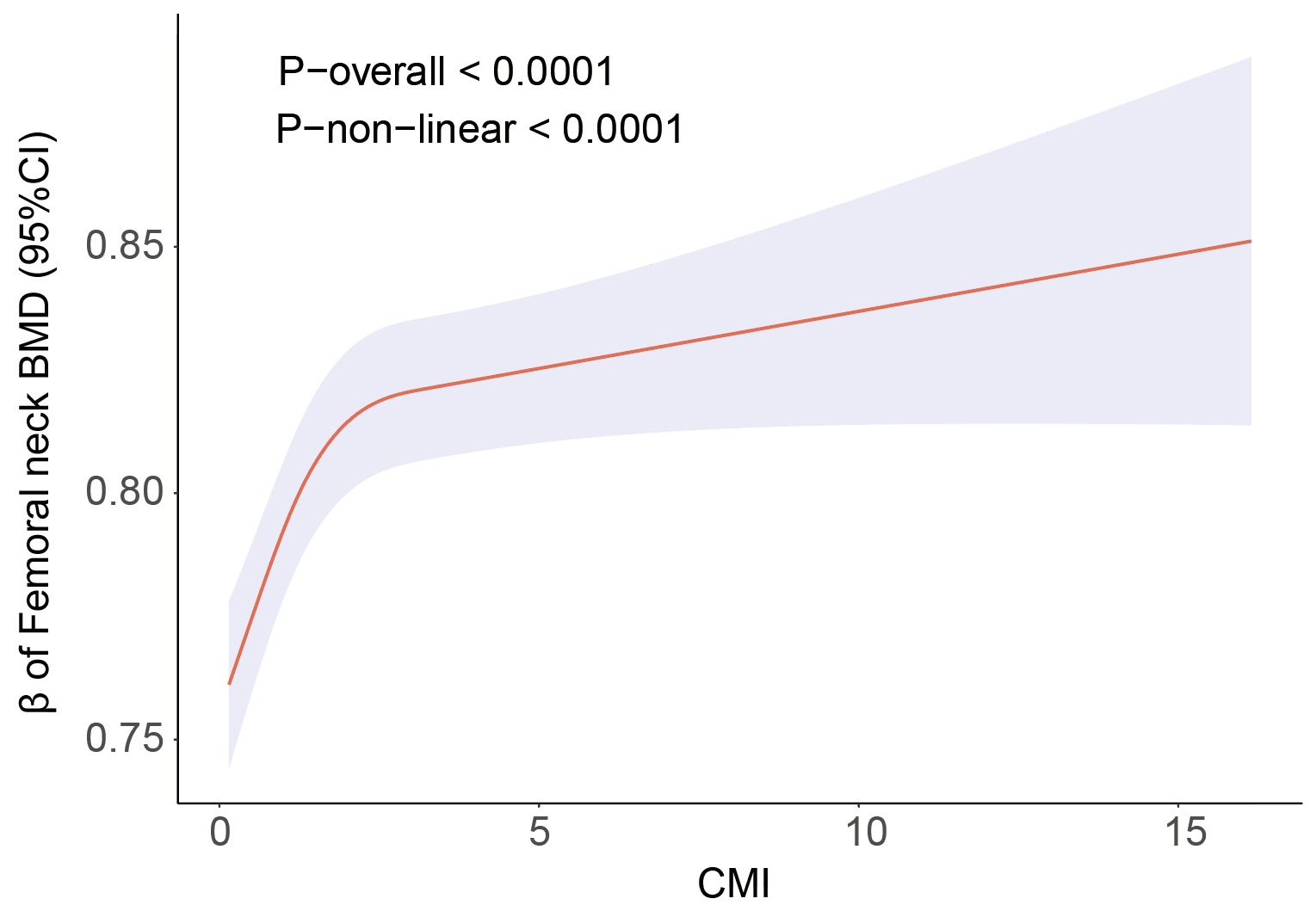


**Supplementary figure 2: RCS analysis fitted the relationship between CMI and Femoral neck BMD.**

Adjusted for age, sex, race, educational level, PIR, calcium, phosphorus, smoke, hypertension, CKD, diabetes, SCR, SUA, AST, ALT.


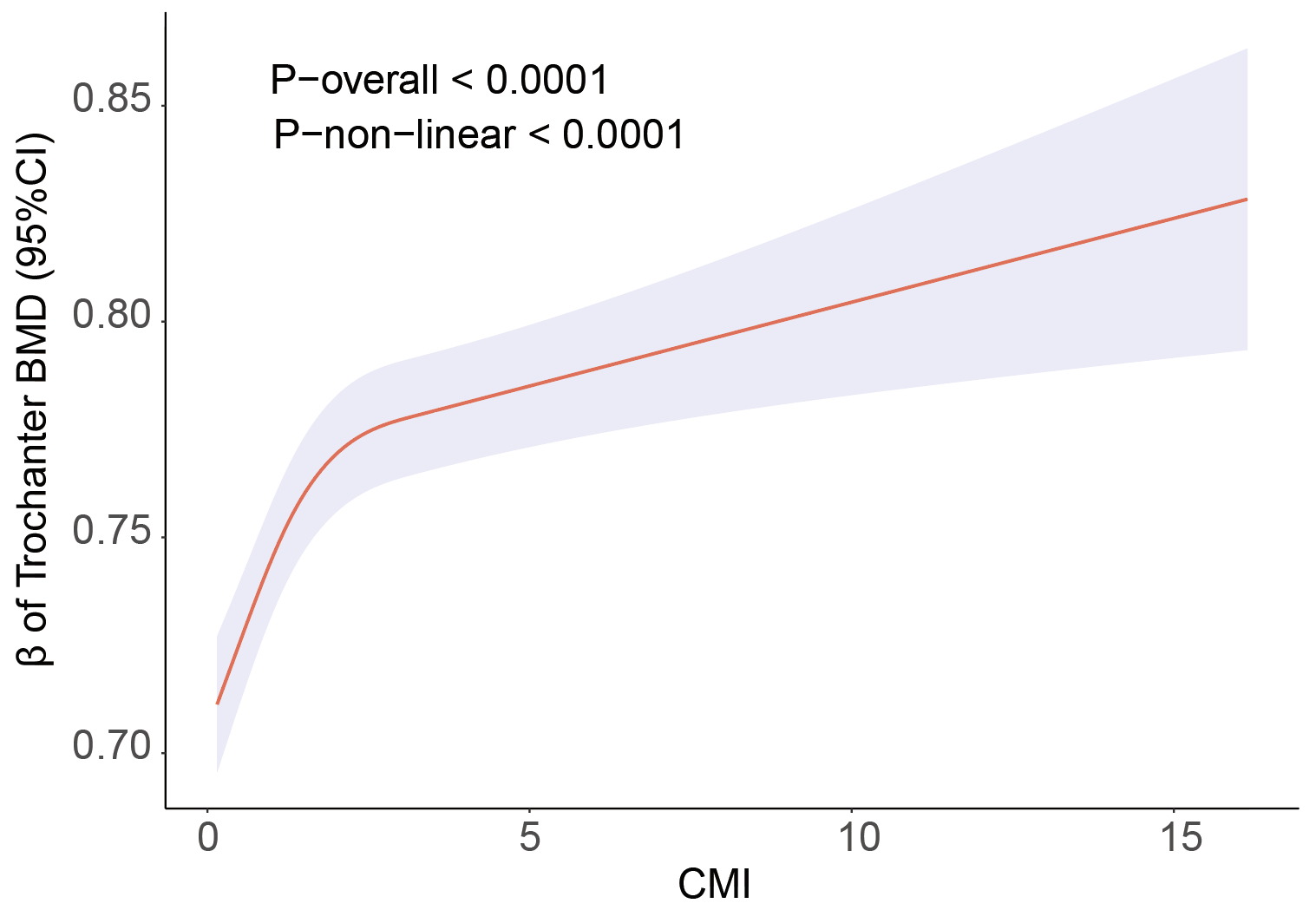
 **Supplementary figure 3: RCS analysis fitted the relationship between CMI and Trochanter BMD.**

Adjusted for age, sex, race, educational level, PIR, calcium, phosphorus, smoke, hypertension, CKD, diabetes, SCR, SUA, AST, ALT.


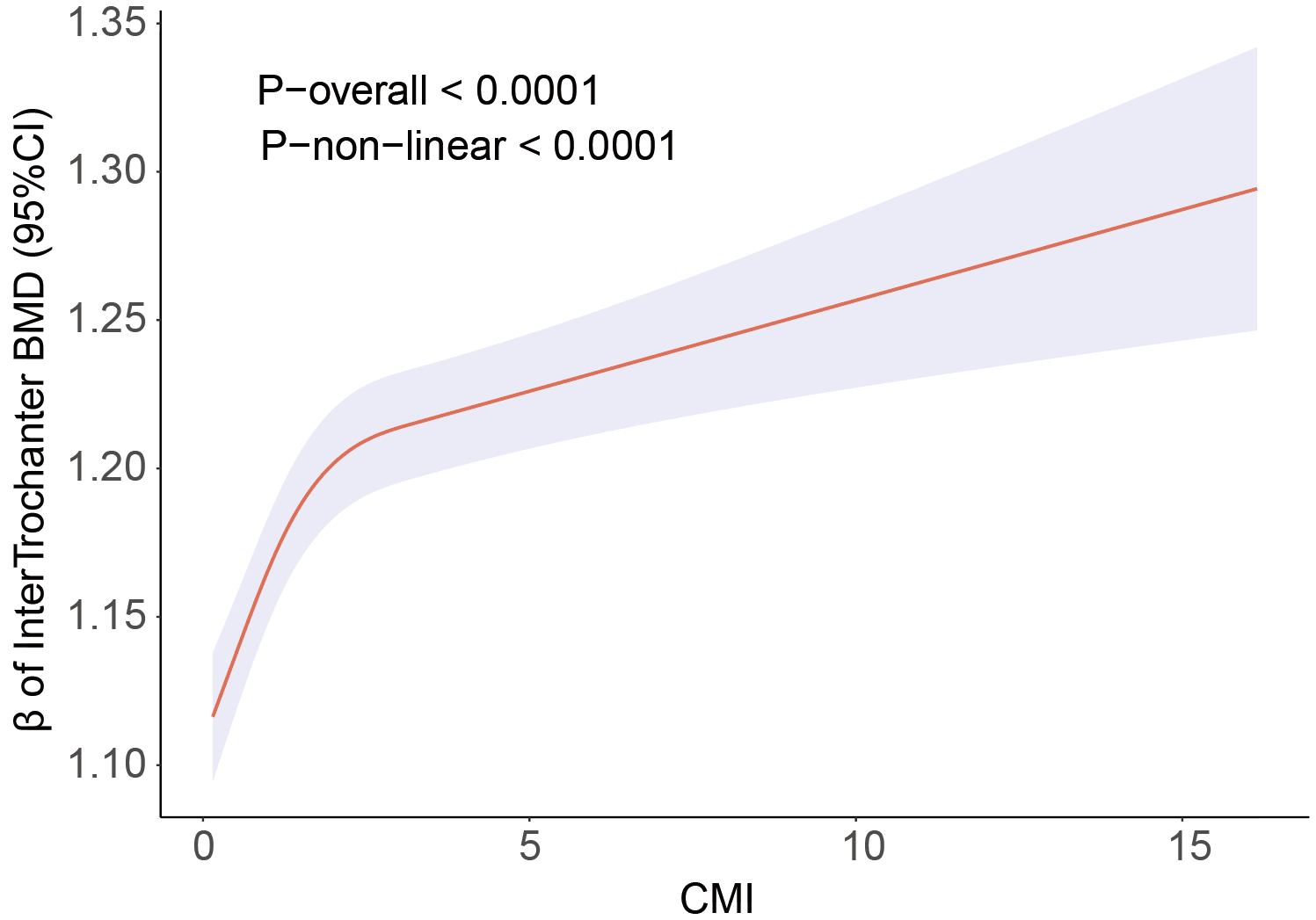
 **Supplementary figure 4: RCS analysis fitted the relationship between CMI and Intertrochanter BMD.**

Adjusted for age, sex, race, educational level, PIR, calcium, phosphorus, smoke, hypertension, CKD, diabetes, SCR, BUN, AST, ALT.
